# Effectiveness of Audiovisual Distraction During Dental Treatment Under Nitrous Oxide/Oxygen Conscious Sedation in Paediatric Patients: A Randomized Crossover Clinical Trial

**DOI:** 10.3390/children13060812

**Published:** 2026-06-12

**Authors:** Tina Gentile, Sonia Vanacore, Martina Caputo, Francesco Pio Litta, Annelyse Martine Garret-Bernardin, Beatrice Basile, Simone Piga, Alessandra Putrino, Angela Galeotti

**Affiliations:** 1 Dentistry Unit, Bambino Gesù Children’s Hospital, IRCCS, Viale Ferdinando Baldelli 41, 00146 Rome, Italy; sonia.vanacore@opbg.net (S.V.); martina.caputo@opbg.net (M.C.); francescopio.litta@opbg.net (F.P.L.); annelyse.garret@opbg.net (A.M.G.-B.); alessandra.putrino@opbg.net (A.P.); angela.galeotti@opbg.net (A.G.); 2 Department of Dental Science, University of Rome Tor Vergata, Via Cracovia 50, 00133 Rome, Italy; 3 Unit of Clinical Epidemiology, Bambino Gesù Children’s Hospital, IRCCS, Piazza Sant’Onofrio 4, 00165 Rome, Italy; epidemiologia@opbg.net

**Keywords:** dental anxiety, conscious sedation, audiovisual distraction, pediatric dentistry

## Abstract

**Highlights:**

**What are the main findings?**

- The combination of audiovisual distraction with nitrous oxide/oxygen conscious sedation is an effective approach for managing dental treatment in anxious paediatric patients.
- The use of audiovisual distraction during dental treatment under nitrous oxide/oxygen conscious sedation enhances the sense of relaxation in paediatric patients.

**What are the implications of the main findings?**

- The association of audiovisual distraction with nitrous oxide/oxygen conscious sedation represents a feasible, safe, and well-accepted strategy for treating uncooperative and phobic paediatric patients.
- Future studies incorporating additional psychophysiological measures (such as skin conductance or salivary cortisol levels) could provide further insight into the interaction between audiovisual distraction and conscious sedation.

**Abstract:**

Background/Objectives: Dental anxiety represents a major challenge in paediatric dentistry and is a frequent cause of non-cooperative behaviour during dental treatment. Nitrous oxide/oxygen inhalation conscious sedation is widely used to reduce anxiety in children, while audiovisual distraction is a non-pharmacological behavioural technique aimed at diverting attention from stressful stimuli. Evidence regarding the combined effect of these two approaches during dental treatment is still limited. Methods: This randomized crossover clinical trial included 25 paediatric patients aged 4–7 years with dental anxiety and previous failed attempts at conventional dental treatment. Each child underwent two dental treatment sessions under nitrous oxide/oxygen conscious sedation, one with and one without audiovisual distraction. Anxiety and behaviour were assessed using the Modified Venham Scale and the Facial Image Scale (FIS). Vital parameters were recorded before, during, and after sedation. Results: A significant reduction in heart rate over time was observed in both groups (*p* < 0.05). In children aged 4–5 years, the combined audiovisual distraction and conscious sedation approach was associated with significantly lower heart rate values compared to conscious sedation alone (*p* < 0.05). No significant differences were found between the two approaches for behavioural scores assessed by the Venham and FIS scales. Conclusions: Although behavioural scores did not differ significantly, audiovisual distraction contributed to greater physiological stability, particularly in terms of heart rate control. This no-pharmacological approach may complement the pharmacological effects of nitrous oxide sedation by enhancing the overall sense of relaxation and comfort during dental care.

## 1. Introduction

Dental anxiety is defined as a persistent and excessive fear of dental stimuli and procedures. Dental anxiety may be caused by fear of potentially painful or invasive procedures, noise produced in the dental clinic, unfamiliar surroundings, parental influence, and previous painful healthcare experiences [1].

The cooperation of children during treatment represents one of the main challenges in paediatric dental care. Children may react differently to the same stimulus according to their temperament, which refers to the innate psychological dispositions and behavioural responses of an individual to internal and external stimuli.

Dental anxiety can increase pain perception in children and is considered one of the main factors triggering non-cooperative behaviours during dental care. When planning a treatment approach for paediatric patients, clinicians must consider several variables, including the patient’s behavioural attitude, sociocultural background, age, oral health status, previous negative dental experiences, and the type of treatment and equipment used. The child’s adaptation to the dental environment can be improved through effective communication strategies and behavioural guidance techniques.

Clinicians can use specific therapies, such as behavioral techniques, to manage dental anxiety. Audiovisual distraction belongs to basic behavioral methods [2]. Audiovisual distraction is defined as a non-aversive approach used to reduce a child’s discomfort by diverting attention away from the main task to accomplish successful, high-quality treatment [3]. Television sets, computer games, and 3D video glasses engage both the visual and auditory components of the patient [1]. These devices are simple and easy to manage.

Basic behavioral techniques are not always effective, especially for highly uncooperative patients. In these cases, clinicians can use additional behavior guidance options, such as nitrous oxide/oxygen inhalation [2].

Nitrous oxide/oxygen inhalation is a safe and effective technique used to reduce anxiety and enhance effective communication. In this technique, the administration of a drug produces a state of depression of the central nervous system, enabling treatment to be carried out while maintaining verbal contact with the patient throughout the period of sedation. The level of sedation must be such that the patient remains conscious, retains protective reflexes, and is able to understand and respond to verbal commands [4].

Previous systematic reviews have demonstrated that nitrous oxide/oxygen inhalation conscious sedation is a safe and effective technique for reducing anxiety and improving cooperation during paediatric dental treatment [5].

Previous studies demonstrated that the use of either sedation techniques or basic behavioral methods alone may not be completely effective in treating uncooperative and phobic paediatric patients [6,7].

A limited number of studies analyzed the combination of sedation techniques and audiovisual distraction during dental treatment in paediatric patients.

The aim of this study was to investigate the effectiveness of sedation techniques (nitrous oxide/oxygen inhalation) and audiovisual distraction during dental treatment on pediatric fearful patients. The hypothesis was that nitrous oxide/oxygen inhalation and audiovisual distraction would facilitate cooperative behaviour and achieve a high level of patient satisfaction during restorative dental treatment.

## 2. Materials and Methods

A randomized clinical trial was conducted at the Dentistry Unit of Bambino Gesù Children’s Hospital, Rome, Italy. This study was performed in accordance with the Declaration of Helsinki and followed the CONSORT 2025 guidelines for randomized crossover trials [8]. In addition, a STROBE checklist/table was included to improve methodological transparency and facilitate the interpretation of participant selection procedures and potential sources of bias (Table 1) [9]. Ethical approval was obtained from the Ethics Committee of Bambino Gesù Childres Hospital, Rome, Italy, and the study protocol was registered under number 2756_OPBG_2022. Verbal and written explanations of the study procedures were provided to the patient’s parents or legal guardians, and written informed consent was obtained prior to enrolment.

The study population consisted of healthy paediatric patients aged 4–7 years presenting with dental anxiety and previous failed attempts at conventional dental treatment. The PICO framework was defined as follows:

Population (P): healthy non-cooperative paediatric patients aged 4–7 years;
Intervention (I): conservative dental treatment under nitrous oxide/oxygen conscious sedation combined with audiovisual distraction;
Comparison (C): conservative dental treatment under nitrous oxide/oxygen conscious sedation alone;
Outcomes (O): behavioural and anxiety outcomes assessed using the Modified Venham Scale and Facial Image Scale (FIS), together with physiological parameters, including oxygen saturation, blood pressure, and heart rate.

The primary outcome of the study was patient cooperation during dental treatment, assessed using behavioural scales and physiological parameters. The secondary outcome was treatment success.

This study was designed as a randomized crossover controlled clinical trial in which each participant underwent two dental treatment sessions under different experimental conditions. Each child served as their own control, thereby minimizing inter-individual variability. No washout period was applied because the interventions consisted of behavioral management approaches without pharmacological residual effects beyond the treatment session. Nevertheless, a possible carry-over effect related to the child’s previous treatment experience cannot be completely excluded and was considered among the study limitations. Carry-over effect was defined as the influence of the experience gained during the first visit on behavioral and physiological responses during the subsequent session [10].

**Figure 1:**
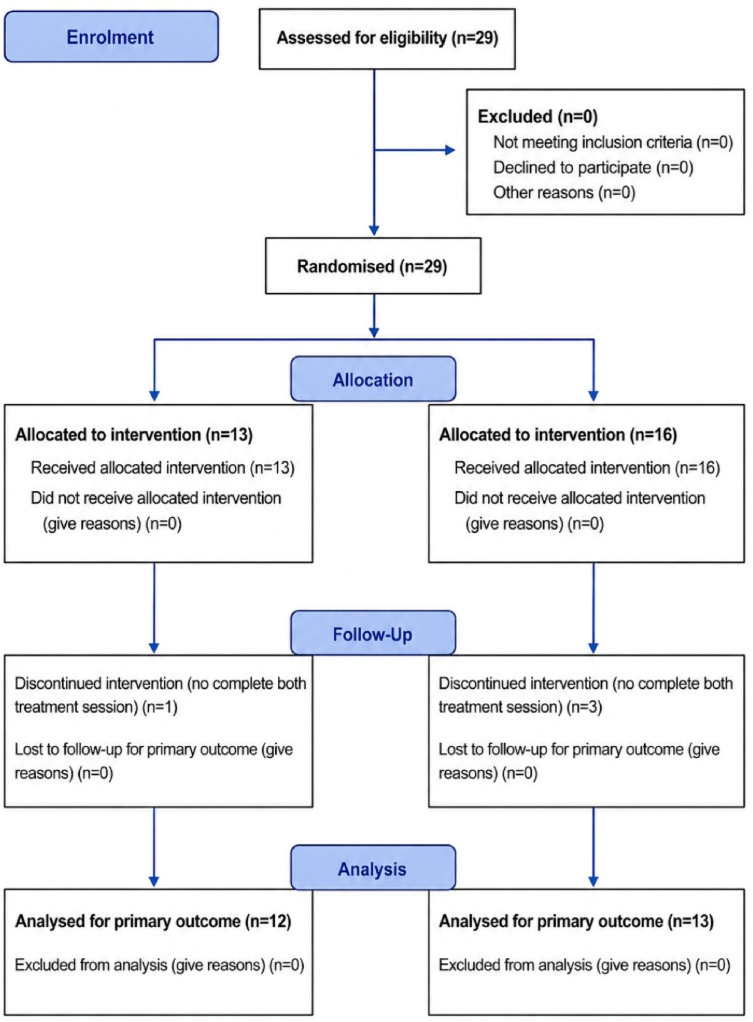
Flow chart of the study design.

Twenty-nine patients were initially assessed for eligibility. Four patients did not complete both treatment sessions and were excluded from the final analysis. Therefore, 25 paediatric patients completed the study and were included in the statistical analysis (Figure 1).

Inclusion criteria (Table 2) were:

- Dental caries requiring conservative treatment;
- Good general health;
- Absence of chronic pharmacological therapy affecting the central nervous system;
- Previous failed attempts at conventional dental treatment.

Exclusion criteria (Table 2) were:

- Age < 4 years or >7 years;
- Systemic diseases;
- Physical or cognitive impairments;
- Ongoing pharmacological therapy;
- Ocular diseases;
- Ear diseases.

The dental treatment consisted of two clinical sessions of conservative dental therapy performed on homologous sites within the same dental arch in order to ensure comparable procedural conditions (Table 3).

Participants were randomly assigned to one of two sequences using a computer-generated randomization list with a 1:1 allocation ratio, permuted blocks of fixed size (four), and stratification by sex. Allocation concealment was ensured using sequentially numbered opaque sealed envelopes prepared by an investigator not involved in patient enrolment or treatment assignment.

An a priori sample size calculation was performed using G*Power software (version 3.1.9.7, Heinrich-Heine-University Düsseldorf, Germany). The calculation was based on the behavioural primary outcome, assessed using the Venham Behaviour Rating Scale. As no pilot data and no directly comparable crossover studies were available at the study planning stage, a standardized effect size of Cohen’s d = 0.6 was assumed as a reasonable estimate of a moderate-to-large treatment effect. Assuming a statistical power of 80%, a two-sided alpha level of 0.05, and paired comparisons within the crossover design, a minimum sample size of 24 participants was estimated. To account for a potential dropout rate of approximately 15%, 29 patients were enrolled.

The two treatment sequences were defined as follows:

- Group 1: first session with nitrous oxide/oxygen conscious sedation alone (A), followed by a second session with conscious sedation combined with audiovisual distraction (B);
- Group 2: first session with conscious sedation combined with audiovisual distraction (B), followed by a second session with conscious sedation alone (A) (Table 3).

Audiovisual distraction consisted of the projection of an age-appropriate cartoon on an electronic display positioned in front of the patient during dental treatment under nitrous oxide/oxygen conscious sedation.

Two cartoons were selected according to the patient’s age: “Finding Nemo” (Disney-Pixar, 2003) for children aged 4–5 years and “Pets” (Universal Pictures, 2016) for children aged 6–7 years. Cartoons were selected according to age appropriateness and emotional content, in order to elicit a positive affective response without interfering with verbal communication between the patient and clinician. In fact, the “funny animals” describe a wide range of animated other-than-human animals with some degree of anthropomorphism. These stylistic choices are often made with the intent to create an affective response from the audience [11].

Conscious sedation was administered using the Master Flux Plus system (Tecno-Gaz S.P.A., Sala Baganza, PR, Italy), with a maximum concentration of 50% nitrous oxide and 50% oxygen, in accordance with international guidelines.

Vital parameters, including oxygen saturation, blood pressure, and heart rate, were monitored before, during, and after conscious sedation. Oxygen saturation and heart rate were continuously monitored, whereas blood pressure values and nitrous oxide concentration were recorded every 10 min. After the procedure, vital signs and possible adverse events, including pain, nausea, and vomiting, were reassessed.

During each appointment, a trained independent observer assessed and recorded the child’s behaviour throughout the dental procedure. Blinding was not feasible because of the nature of the intervention. Behavioural assessment was performed at the following time point:

– Tc: first contact with the dentist;
– T0: start of sedation induction;
– T1: end of induction (after 8–10 min);
– T2: administration of local anesthesia, when required;
– T3: during dental treatment under conscious sedation with or without the audiovisual distraction;
– T4: end of the session.

According to the Modified Venham Scale, scores from 0 (relaxed child) to 5 (out of control) were assigned to each phase to evaluate patient cooperation (Table 4) [12,13].

**Figure 2:**
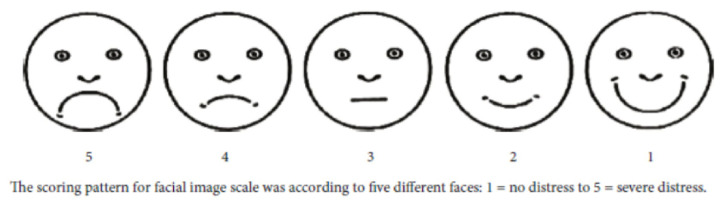
Facial Image Scale (FIS).

At Tc and T4, anxiety was additionally assessed using the Facial Image Scale (FIS), which includes five facial expressions ranging from very happy (score 1) to very unhappy (score 5) (Figure 2) [14].

Categorical variables were summarized as absolute frequencies and percentages, whereas continuous variables were expressed as means and standard deviations. The Shapiro–Wilk test was used to assess data normality.

Given the crossover design of the study, each participant served as their own control. Behavioural and physiological parameters obtained under the two treatment conditions were therefore analysed using within-subject comparisons across the different observation time points.

Changes over time within each treatment condition and differences between treatment conditions were evaluated according to data distribution and the repeated-measures structure of the study design.

For continuous variables with normal distribution, paired t-tests were used for within-subject comparisons between the two treatment conditions. When data were not normally distributed, non-parametric analyses were applied, as appropriate. Categorical variables were analysed using the χ2 test or Fisher’s exact test. The analysis focused on behavioural scores and physiological trends recorded during the two treatment sessions.

Statistical analyses were conducted using STATA software (Release 13; StataCorp LP, College Station, TX, USA). Statistical significance was set at *p* < 0.05.

## 3. Results

### 3.1. Participant Characteristics

Of the 29 patients initially enrolled, four did not complete both treatment sessions and were therefore excluded from the final analysis. The study sample consequently consisted of 25 pediatric patients, aged 4–7 years (Table 5).

No locoregional anesthesia was administered in either treatment condition.

### 3.2. Physiological Parameters

Analysis of the physiological parameters showed that heart rate was the only variable demonstrating significant changes over time. In both treatment conditions, heart rate progressively decreased from baseline to the operative phases of treatment. In the audiovisual distraction plus conscious sedation condition, significant correlations between heart rate and nitrous oxide concentration were observed at T0 (r = 0.691, *p* < 0.001), T1 (r = 0.756, *p* < 0.001), and T3 (r = 0.586, *p* = 0.002). Similarly, in the conscious sedation-only condition, heart rate was significantly correlated at T0 (r = 0.602, *p* = 0.002), T1 (r = 0.591, *p* = 0.002), and T3 (r = 0.620, *p* = 0.001). Within-subject comparisons between the two treatment conditions showed lower heart rate values during the audiovisual distraction plus conscious sedation sessions, particularly in younger children (4–5 years old).

No significant differences or associations were observed for systolic blood pressure, diastolic blood pressure, or oxygen saturation in either treatment condition (Table 6).

### 3.3. Age-Stratified Analysis

When data were stratified by age, a more pronounced effect was observed in younger children (4–5 years). In this subgroup, significant correlations for heart rate were detected only in the audiovisual distraction plus conscious sedation condition at T0 (r = 0.556, *p* = 0.025), T1 (r = 0.744, *p* = 0.001), and T3 (r = 0.515, *p* = 0.041). No significant correlations were observed in the conscious sedation-only condition (Table 7).

Among patients aged 6–7 years, heart rate was significantly associated with nitrous oxide concentration in both treatment conditions. In the audiovisual distraction plus conscious sedation condition, significant correlations were observed at T0 (r = 0.556, *p* = 0.025), T1 (r = 0.784, *p* = 0.012), and T3 (r = 0.920, *p* < 0.001). In the conscious sedation-only condition, heart rate was significantly correlated at T1 (r = 0.842, *p* = 0.004) and T3 (r = 0.887, *p* = 0.001). Blood pressure and oxygen saturation did not show significant changes in either age group or treatment condition (Table 8).

### 3.4. Gender Differences

No statistically significant differences were found between male and female patients for any clinical parameter in either treatment condition (Table 9).

### 3.5. Relationship Between Physiological Parameters and Behaviour

Furthermore, no significant correlations were detected between clinical parameters and Venham behavioural scores in either treatment approach (Table 10).

### 3.6. Relationship Between Venham and FIS Score

However, a significant positive correlation between Venham and FIS scores was observed in the conscious sedation-only condition, both at baseline (Tc: r = 0.489, *p* = 0.013) and at the end of treatment (T4: r = 0.543 and *p* = 0.005).

In the audiovisual distraction plus conscious sedation condition, a significant correlation was observed only at baseline (Tc: r = 0.477, *p* = 0.016), with no association at T4 (Table 11).

## 4. Discussion

Dental anxiety is a state of apprehension that something dreadful may occur during dental treatment, often resulting in a perceived loss of control [15].

This emotional condition is considered one of the main sources of behaviour management difficulties in paediatric dentistry and can hinder the delivery of high-quality dental care.

Distraction techniques can suppress pain-related nervous system activity by providing competing sensory stimuli, potentially producing analgesic effects through the release of endogenous opioids at opioid receptor sites. Activation of opioid receptors at the interneuronal level within the spinal cord results in neuronal hyperpolarization and inhibition of pain transmission. The periaqueductal grey (PAG) region plays a central role in modulating descending pain control pathways that inhibit nociceptive information at the spinal cord level. The experience of pain is processed across multiple brain regions, with the somatosensory cortex (S1) and the affective-emotional dimension involving the anterior cingulate cortex (ACC) [16].

Watching animated cartoon videos as a form of audiovisual distraction may divert the child’s attention away from nociceptive and anxiety-provoking stimuli, thereby reducing fear and anxiety and potentially minimizing the perception of trauma associated with dental procedures [17]. In this context, audiovisual distraction should not be considered merely an accessory entertainment tool, but rather an integral component of behavioral guidance during conscious sedation procedures. For this reason, adequate communication with parents and caregivers before treatment is essential. During the essential informed consent process, families should be informed not only about the pharmacological aspects and safety profile of conscious sedation [18], but also about the role of behavioural support strategies, including audiovisual distraction, in improving the child’s emotional comfort, cooperation, and overall treatment experience. Increasing parental awareness of these supportive approaches may facilitate acceptance of the procedure and contribute to a more positive dental experience for the child [19].

**Figure 3:**
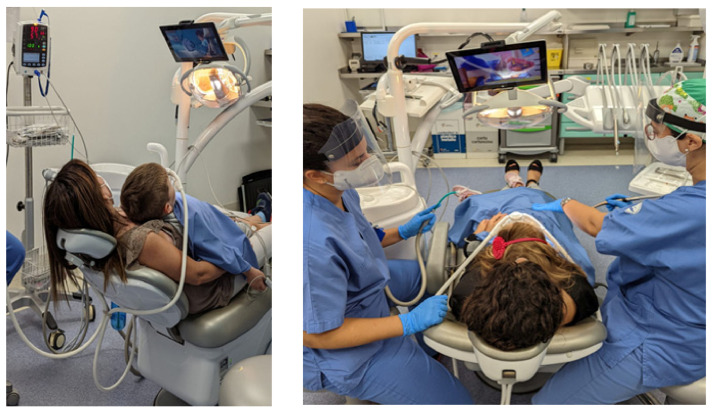
Use of audiovisual distraction during dental treatment under conscious sedation.

Furthermore, recent technological advances, including eye-tracking systems and visual attention analysis, also applied to the dental setting, have demonstrated how strongly the human brain can be influenced and absorbed by visual stimuli, often without conscious awareness of such conditioning [20,21]. These findings support the neuropsychological basis of audiovisual distraction techniques and reinforce the importance of visual engagement in modulating attention, emotional responses, and pain perception during dental treatment. In the present randomized crossover clinical trial, we evaluated the effectiveness of audiovisual distraction combined with conscious sedation with nitrous oxide and oxygen during dental treatment in anxious pediatric patients (Figure 3).

Our results demonstrated that both treatment approaches enabled completion of dental procedures. Although audiovisual distraction was associated with changes in physiological parameters, particularly heart rate, no significant differences were observed in the main behavioural outcomes assessed by the Venham and FIS scales. Therefore, the clinical relevance of heart rate modulation alone should be interpreted cautiously. The observed reduction in heart rate may suggest a lower autonomic stress response during treatment; however, this physiological finding did not correspond to measurable improvements in behavioural cooperation or self-reported anxiety scores. Moreover, given the relatively small sample size and the multiple correlation analyses performed, these findings should be considered exploratory rather than definitive.

The mechanisms underlying the effects of audiovisual distraction may involve reduced sympathetic nervous system stimulation, inhibition of sympathetic activity, and decreased release of stress-related mediators, thereby preventing increases in heart rate and contributing to physiological stability [22,23,24].

These mechanisms may also be explained, at least in part, by the “gate control theory” of pain modulation, according to which competing sensory and cognitive stimuli may reduce the central processing of nociceptive information [25]. In paediatric dentistry, audiovisual distraction may therefore act by engaging the child’s attentional and emotional resources, limiting the brain’s perception of painful or anxiety-provoking stimuli during treatment.

This aspect may be particularly relevant during procedures requiring local anaesthetic injections [26]. In these situations, simple manual distraction techniques, such as vibration, cold devices, or basic behavioural redirection [27,28], as well as pharmacological premedication alone, may not always be sufficient to achieve an effective attentional shift away from the invasive stimulus, especially in younger children [29]. By contrast, immersive audiovisual stimulation may provide a more effective cognitive and sensory distraction capable of modulating the child’s emotional and physiological response during dental procedures [30,31,32].

In the present study, this effect was particularly evident in younger children aged 4–5 years, who are generally characterized by higher emotional reactivity and a lower capacity for cognitive self-regulation during stressful situations such as dental treatment [33].

Conversely, the absence of significant differences in behavioural scores between the two treatment approaches may be explained by the strong anxiolytic effect of nitrous oxide sedation itself. As previously reported, nitrous oxide alone is associated with high success rates and satisfactory patient cooperation in pediatric dentistry [4,13,34]. This pharmacological effect may have produced a ceiling effect, limiting the detection of additional behavioural improvements attributable to audiovisual distraction.

In a systematic review, Zhang et al. (2019) reported that the audiovisual content delivered through immersive eyeglasses may have a stronger impact than content displayed on conventional screens [1].

However, in the present study, audiovisual eyeglasses were not used because continuous verbal interaction with the child was considered essential during conscious sedation with nitrous oxide and oxygen.

Nitrous oxide/oxygen inhalation induces a mild depression of the central nervous system while preserving consciousness, protective reflexes, and responsiveness to verbal commands. Highly immersive audiovisual devices might interfere with the clinician’s ability to continuously assess the child’s level of consciousness, making them less suitable in this specific clinical context [35].

Our findings partially agree with Zhang et al. (2019), who demonstrated significant reductions in anxiety and pain perception using audiovisual distraction alone during dental treatment [1,36,37].

However, unlike previous studies, our protocol combined audiovisual distraction with pharmacological sedation, which may have reduced the magnitude of observable behavioural differences between experimental conditions.

The absence of a treatment arm using audiovisual distraction alone represents an important limitation of the present study. However, all included patients had previously demonstrated dental anxiety and unsuccessful conventional treatment attempts; therefore, withholding conscious sedation was considered ethically and clinically inappropriate in this population. Consequently, the study design does not allow the independent effect of audiovisual distraction to be fully distinguished from the anxiolytic effect of nitrous oxide/oxygen sedation.

No significant associations were found between behavioural scores and physiological parameters, suggesting that behavioural scales and vital signs may represent distinct and complementary components of the child’s stress response [38]. Nevertheless, the significant correlation observed between Venham and FIS scores in the conscious sedation group at both baseline and end of treatment supports the internal consistency of these tools for assessing pediatric dental anxiety.

No significant differences were observed between male and female participants for any of the clinical or behavioural variables considered.

This study has several limitations. First, despite the crossover design reducing inter-individual variability, the relatively small sample size may have limited the ability to detect smaller-than-anticipated effects, particularly for the behavioural outcome assessed by the Venham Behaviour Rating Scale. Although this study was powered a priori to detect a standardized effect size of Cohen’s d = 0.6, smaller but potentially clinically relevant differences may have remained undetected, thereby limiting the generalizability of the findings. In addition, the multiple correlation analyses performed should be interpreted cautiously due to the increased risk of type I error. Furthermore, possible learning or adaptation effects between treatment sessions cannot be completely excluded. Behavioural assessment was conducted by a single observer, which may have introduced observer-related bias despite standardized training. Future studies with larger samples, additional psychophysiological measures, and parallel comparison groups are needed to better clarify the independent contribution of audiovisual distraction during paediatric dental sedation [39,40].

Despite these limitations, this study suggests that combining audiovisual distraction with nitrous oxide/oxygen sedation is a feasible, safe, and well-accepted strategy. Even in the absence of significant behavioural differences, the observed physiological stabilization indicates a potential synergistic calming effect, which may be particularly relevant in younger or highly anxious children. From a clinical perspective, integrating audiovisual distraction into sedation protocols may enhance patient comfort and cooperation, facilitating treatment success and potentially improving future dental experiences.

## 5. Conclusions

This randomized crossover clinical trial suggests that audiovisual distraction may represent a useful adjunctive strategy during dental treatment performed under nitrous oxide/oxygen conscious sedation in anxious paediatric patients. Although no significant differences were observed in behavioural outcomes assessed using the Venham and FIS scales, audiovisual distraction was associated with changes in physiological parameters, particularly heart rate modulation. However, the clinical significance of these findings remains uncertain. Due to the absence of a non-sedated comparison group and the relatively small sample size, the results should be interpreted cautiously. Further studies with larger populations and additional control groups are required to better clarify the independent contribution of audiovisual distraction during paediatric dental treatment.

## Figures and Tables

**Table 1:** STROBE table.

Strobe Item	Description in the Present Study
Study design	Randomized crossover controlled clinical trial
Participants	Twenty-nine paediatric patients were initially enrolled; 25 children, aged 4–7 years, completed both treatment sessions and were included in the final analysis. All patients had dental anxiety and previous failed attempts at conventional dental treatment.
Variables	Behavioural outcomes (Modified Venham Scale, FIS) and physiological parameters were defined.
Data sources/measurement	Continuous monitoring of heart rate and oxygen saturations; validated behavioural scale used.
Bias	Randomization and crossover design reduced inter-individual variability.
Study size	A priori sample size calculation was performed using G*Power software.
Quantitative variables	Continuous variables expressed as mean ± SD.
Statistical methods	- The Shapiro–Wilk test was used. Comparisons between categorical variables were performed using the χ^2^ test or Fisher’s exact test. Continuous variables were analyzed using a paired *t*-test. - Age-stratified and gender analyses performed. - Dropout mentioned.

**Table 2:** Inclusion/exclusion criteria.

Inclusion Criteria	Exclusion Criteria
Age range: 4–7 years	Age ˂ 4 years; Age ˃ 7 years
Dental caries requiring conservative treatment	Systemic diseases (physical or cognitive impairments)
Good general health	Ocular diseases
Previous failed attempts at conventional dental treatment	Ear diseases
Absence of chronic pharmacological therapy affecting the central nervous system	Ongoing pharmacological therapy

**Table 3:** Study group allocation.

Group Allocation	Treatment Sequence
Group 1	First session: conscious sedation using nitrous oxide and oxygen (A). Second session: conscious sedation combined with audiovisual distraction (B).
Group 2	First session: conscious sedation combined with audiovisual distraction (B). Second session: conscious sedation alone (A).

**Table 4:** Modified Venham Scale [13].

0. Relaxed: smiling, willing, able to converse, displays behavior desired by the dentist.
1. Uneasy: concerned, may protest briefly to indicate discomfort, hands remain down or partially raised. Tense facial expression, high chest. Capable of cooperating.
2. Tense: tone of voice, question and answers reflect anxiety. During stressful procedure, verbal protest, crying, hands tensed and raised, but not interfering very much. Protest more distracting and troublesome. Child still complies with the request to cooperate.
3. Reluctant: pronounced verbal protest, crying. Using hands to stop procedure. Treatment proceeds with difficulty
4. Interference: general crying, body movements sometimes needing physical restraint. Protest disrupts procedure.
5. Out of contact: hard loud crying, swearing, screaming. Unable to listen, trying to escape. Physical restraint require.

**Table 5:** Participant characteristics.

N.	25
Age (mean ± SD)	5 ± 1.3
Gender (%)	M = 52; F = 48

**Table 6:** Pearson’s correlations by clinical parameters and type of treatment.

Type of Treatment and Clinical Parameters										
	Timeline	*p*-Value	Timeline	*p*-Value	Timeline	*p*-Value	Timeline	*p*-Value	Timeline	*p*-Value
Audiovisual approach and conscious sedation	T0		T1		T2		T3		T4	
Systolic blood pressure	0.302	0.161	−0.097	0.646	n.r.	-	0.173	0.408	n.r.	-
Diastolic blood pressure	0.205	0.349	0.333	0.103	n.r.	-	0.360	0.077	n.r.	-
Heart rate	0.691	<0.001	0.756	<0.001	n.r.	-	0.586	0.002	n.r.	-
Oxygen saturation	0.074	0.737	−0.177	0.396	n.r.	-	0.028	0.895	n.r.	-
Conscious sedation										
Systolic blood pressure	0.121	0.567	−0.024	0.908	n.r.	-	0.058	0.782	n.r.	-
Diastolic blood pressure	0.253	0.223	−0.067	0.751	n.r.	-	−0.204	0.329	n.r.	-
Heart rate	0.602	0.002	0.591	0.002	n.r.	-	0.620	0.001	n.r.	-
Oxygen saturation	−0.262	0.206	−0.155	0.460	n.r.	-	0.072	0.732	n.r.	-

n.r.: not recorded.

**Table 7:** Pearson’s correlations by clinical parameters and type of treatment in patients aged 4–5 years.

Type of Treatment and Clinical Parameters										
	Timeline	*p*-Value	Timeline	*p*-Value	Timeline	*p*-Value	Timeline	*p*-Value	Timeline	*p*-Value
Audiovisual approach and conscious sedation	T0		T1		T2		T3		T4	
Systolic blood pressure	0.240	0.371	−0.069	0.799	n.r.	-	0.012	0.965	n.r.	-
Diastolic blood pressure	0.029	0.915	0.310	0.243	n.r.	-	0.369	0.159	n.r.	-
Heart rate	0.556	0.025	0.744	0.001	n.r.	-	0.515	0.041	n.r.	-
Oxygen saturation	0.086	0.809	−0.343	0.194	n.r.	-	−0.324	0.221	n.r.	-
Conscious sedation										
Systolic blood pressure	0.270	0.313	0.107	0.694	n.r.	-	−0.049	0.857	n.r.	-
Diastolic blood pressure	0.173	0.522	0.123	0.651	n.r.	-	−0.133	0.624	n.r.	-
Heart rate	0.292	0.273	0.088	0.746	n.r.	-	0.216	0.423	n.r.	-
Oxygen saturation	0.188	0.485	<0.001	1.000	n.r.	-	0.304	0.253	n.r.	-

n.r.: not recorded.

**Table 8:** Pearson’s correlations by clinical parameters and type of treatment in patients aged 6–7 years.

Type of Treatment and Clinical Parameters										
	Timeline	*p*-Value	Timeline	*p*-Value	Timeline	*p*-Value	Timeline	*p*-Value	Timeline	*p*-Value
Audiovisual approach and conscious sedation	T0		T1		T2		T3		T4	
Systolic blood pressure	0.132	0.778	0.018	0.964	n.r.	-	0.249	0.518	n.r.	-
Diastolic blood pressure	0.238	0.607	0.419	0.262	n.r.	-	0.206	0.595	n.r.	-
Heart rate	0.556	0.025	0.784	0.012	n.r.	-	0.920	<0.001	n.r.	-
Oxygen saturation	*		*		n.r.	-	0.553	0.122	n.r.	-
Conscious sedation										
Systolic blood pressure	0.112	0.776	0.139	0.722	n.r.	-	0.022	0.955	n.r.	-
Diastolic blood pressure	0.112	0.776	−0.339	0.372	n.r.	-	−0.505	0.166	n.r.	-
Heart rate	0.292	0.273	0.842	0.004	n.r.	-	0.887	0.001	n.r.	-
Oxygen saturation	−0.657	0.055	*		n.r.	-	−0.163	0.675	n.r.	-

*: Correlation coefficient could not be computed (insufficient variability).

**Table 9:** Comparison of vital signs (mean, SD) by gender in two different therapeutic approaches.

Type of Treatment and Clinical Parameters										
	Timeline	*p*-Value	Timeline	*p*-Value	Timeline	*p*-Value	Timeline	*p*-Value	Timeline	*p*-Value
Audiovisual approach and conscious sedation	T0		T1		T2		T3		T4	
Systolic blood pressure		0.928		0.199		0.325		0.567		0.739
M (mean, SD)	102.46 (8.08)		103.07 (6.40)		100.61 (5.24)		104.30 (7.93)		101.69 (4.59)	
F (mean, SD)	102.75 (7.66)		107.16 (8.96)		103.41 (8.45)		102.66 (5.94)		100.83 (7.84)	
Diastolic blood pressure		0.759		0.398		0.336		0.749		0.299
M (mean, SD)	59.53 (8.43)		59.30 (6.03)		60.23 (6.83)		60.92 (8.12)		60.61 (5.91)	
F (mean, SD)	60.67 (9.75)		62.08 (9.79)		62.83 (6.38)		61.83 (5.56)		64.25 (10.71)	
Heart rate		0.669		0.665		0.192		0.849		0.614
M (mean, SD)	85.84 (14.25)		83.00 (9.68)		82.53 (10.08)		86.61 (11.33)		86.15 (12.82)	
F (mean, SD)	83.58 (11.60)		84.58 (8.28)		87.33 (7.43)		85.83 (8.59)		88.58 (10.76)	
Oxygen saturation		0.641		0.156		0.606		0.403		0.704
M (mean, SD)	99.84 (0.38)		99.92 (0.28)		99.84 (0.38)		99.84 (0.38)		99.46 (0.78)	
F (mean, SD)	99.75 (0.62)		99.50 (1.00)		99.91 (0.288)		99.66 (0.65)		99.33 (0.88)	
Conscious sedation										
Systolic blood pressure		0.162		0.628		0.556		0.691		0.657
M (mean, SD)	100.00 (7.35)		99.07 (7.05)		101.15 (8.11)		102.38 (6.77)		104.38 (6.46)	
F (mean, SD)	104.08 (6.75)		100.41 (6.53)		102.83 (5.59)		101.33 (6.23)		103.33 (5.07)	
Diastolic blood pressure		0.197		0.651		0.237		0.682		0.855
M (mean, SD)	60.38 (4.29)		62.38 (6.05)		64.84 (7.09)		63.07 (5.01)		63.07 (7.62)	
F (mean, SD)	64.00 (8.74)		63.66 (7.90)		61.58 (6.27)		62.16 (5.94)		62.58 (5.48)	
Heart rate		0.732		0.240		0.223		0.342		0.493
M (mean, SD)	88.38 (14.56)		88.15 (11.04)		88.30 (11.85)		88.15 (11.69)		85.30 (12.57)	
F (mean, SD)	90.33 (13.46)		82.50 (12.41)		83 (8.98)		83.75 (10.94)		89.25 (15.64)	
Oxygen saturation		0.698		0.934		0.896		0.233		0.154
M (mean, SD)	99.85 (0.55)		99.84 (0.38)		99.69 (0.48)		99.84 (0.38)		99.61 (0.65)	
F (mean, SD)	99.92 (0.29)		99.83 (0.39)		99.66 (0.49)		99.58 (0.67)		99.91 (0.29)	

**Table 10:** Correlation between clinical parameters and Venham score in two different therapeutic approaches.

Type of Treatment and Clinical Parameters												
	Timeline	*p*-Value	Timeline	*p*-Value	Timeline	*p*-Value	Timeline	*p*-Value	Timeline	*p*-Value	Timeline	*p*-Value
Audiovisual approach and conscious sedation	Tc		T0		T1		T2		T3		T4	
Systolic blood pressure	0.265	0.200	0.138	0.511	−0.060	0.773	n.r.	-	−0.169	0.418	0.235	0.256
Diastolic blood pressure	0.020	0.922	−0.054	0.794	0.333	0.103	n.r.	-	0.196	0.348	−0.308	0.134
Heart rate	0.085	0.686	0.232	0.263	0.227	0.274	n.r.	-	−0.208	0.318	−0.061	0.771
Oxygen saturation	−0.134	0.522	−0.050	0.811	−0.083	0.693	n.r.	-	−0.167	0.422	0.000	1.000
Conscious sedation												
Systolic blood pressure	−0.279	0.1769	−0.267	0.196	−0.227	0.274	n.r.	-	−0.201	0.334	0.099	0.637
Diastolic blood pressure	−0.213	0.3051	−0.143	0.493	0.272	0.188	n.r.	-	0.071	0.733	−0.009	0.963
Heart rate	0.015	0.9430	−0.067	0.749	0.074	0.722	n.r.	-	−0.045	0.829	−0.010	0.959
Oxygen saturation	−0.105	0.6158	0.155	0.458	−0.095	0.652	n.r.	-	−0.081	0.610	−0.032	0.876

**Table 11:** Correlation between Venham and FIS based on the type of therapeutic approach.

Type of Treatment	Venham Tc vs. Fis Tc	*p*-Value	Venham t4 vs. Fis t4	*p*-Value
Audiovisual approach and conscious sedation	0.477	0.016	0.117	0.578
Conscious sedation	0.489	0.013	0.543	0.005

## Data Availability

The datasets presented in this article are not readily available because they contain sensitive patient information and are subject to institutional data protection regulations. Requests to access the datasets should be directed to the corresponding author.

## Abbreviations

The following abbreviations are used in this manuscript:

IRCCS	Istituto di Ricovero e Cura a Carattere Scientifico
FIS	Facial Image Scale
PAG	Periaqueductal grey region
S1	Somatosensory cortex
ACC	Anterior cingulate cortex

## References

[1] Zhang C., Qin D., Shen L., Ji P., Wang J. (2019). Does audiovisual distraction reduce dental anxiety in children under local anesthesia? A systematic review and meta-analysis. Oral Dis..

[2] American Academy of Pediatric Dentistry (2025). Behavior guidance for the pediatric dental patient. The Reference Manual of Pediatric Dentistry.

[3] Al-Khotani A., Bello L.A., Christidis N. (2016). Effects of audiovisual distraction on children’s behaviour during dental treatment: A randomized controlled clinical trial. Acta Odontol. Scand..

[4] Galeotti A., Garret A.B., D’Antò V., Ferrazzano G.F., Gentile T., Viarani V., Cassabgi G., Cantile T. (2016). Inhalation Conscious Sedation with Nitrous Oxide and Oxygen as Alternative to General Anesthesia in Precooperative, Fearful, and Disabled Pediatric Dental Patients: A Large Survey on 688 Working Sessions. BioMed Res. Int..

[5] Rossit M., Gil-Manich V., Ribera-Uribe J.M. (2021). Success rate of nitrous oxide-oxygen procedural sedation in dental patients: Systematic review and meta-analysis. J. Dent. Anesth. Pain Med..

[6] Özen B., Malamed S.F., Cetiner S., Özalp N., Özer L., Altun C. (2012). Outcomes of moderate sedation in paediatric dental patients. Aust. Dent. J..

[7] Baygin O., Bodur H., Isik B. (2010). Effectiveness of premedication agents administered prior to nitrous oxide/oxygen. Eur. J. Anaesthesiol..

[8] Hopewell S., Chan A.W., Collins G.S., Hróbjartsson A., Moher D., Schulz K.F., Tunn R., Aggarwal R., Berkwits M., Berlin J.A. (2025). CONSORT 2025 statement: Updated guideline for reporting randomised trials. BMJ.

[9] Cuschieri S. (2019). The STROBE guidelines. Saudi J. Anaesth..

[10] Asvanund Y., Mitrakul K., Juhong R., Arunakul M. (2015). Effect of audiovisual eyeglasses during local anesthesia injections in 5- to 8-year-old children. Quintessence Int..

[11] Dydynski J.M., Mäekivi N. (2021). Impacts of Cartoon Animals on Human–Alloanimal Relations. Anthrozoös.

[12] Venham L.L., Gaulin-Kremer E., Munster E., Bengston-Audia D., Cohan J. (1980). Interval rating scales for children’s dental anxiety and uncooperative behavior. Pediatr. Dent..

[13] Garret A.B., Festa P., Matarazzo G., Vinereanu A., Aristei F., Gentile T., Piga S., Bendinelli E., Cagetti M.G., Galeotti A. (2023). Behavioral Modifications in Children after Repeated Sedation with Nitrous Oxide for Dental Treatment: A Retrospective Study. Int. J. Environ. Res. Public Health.

[14] Buchanan N., Niven N. (2002). Validation of a Facial Image Scale to assess child dental anxiety. Int. J. Paediatr. Dent..

[15] Klingberg G., Broberg A.G. (2007). Dental fear/anxiety and dental behaviour management problems in children and adolescents: A review of prevalence and concomitant psychological factors. Int. J. Paediatr. Dent..

[16] Ramdhanie G.G., Nurrohmah A., Mulya A.P., Mediani H.S., Sumarni N., Mulyana A.M., Huda M.H. (2024). A Scoping Review of Audiovisual Distraction Techniques Among Children in Reducing Invasive Procedure Pain. J. Multidiscip. Healthc..

[17] Ghadimi S., Estaki Z., Rahbar P., Shamshiri A.R. (2018). Effect of visual distraction on children’s anxiety during dental treatment: A crossover randomized clinical trial. Eur. Arch. Paediatr. Dent..

[18] Galeotti A., Putrino A., Garret-Bernardin A.M., Caputo M., Vanacore S., Lodise M., Siodambro C., Grossi G.B., Pellegrini M., Zanette G. (2026). Procedural sedation in dentistry: A scoping review and proposal for an ad hoc informed consent. BMC Med. Ethics.

[19] Al-Halabi M.N., Bshara N., AlNerabieah Z. (2018). Effectiveness of audio-visual distraction using virtual reality eyeglasses versus tablet device in child behavioral management during inferior alveolar nerve block. Anaesth. Pain Intensive Care.

[20] Celine G.R., Cho V.V.Y., Kogan A., Anthonappa R.P., King N.M. (2021). Eye-tracking in dentistry: What do children notice in the dental operatory?. Clin. Oral Investig..

[21] Putrino A., Marinelli E., Raso M., Calace V., Zaami S. (2023). Clear Aligners and Smart Eye Tracking Technology as a New Communication Strategy between Ethical and Legal Issues. Life.

[22] Jain A.A., Yeluri R., Munshi A.K. (2012). Measurement and assessment of pain in children—A review. J. Clin. Pediatr. Dent..

[23] Brand H.S., Abraham-Inpijn L. (1996). Cardiovascular responses induced by dental treatment. Eur. J. Oral Sci..

[24] AlMaummar M., AlThabit H.O., Pani S. (2019). The impact of dental treatment and age on salivary cortisol and alpha-amylase levels of patients with varying degrees of dental anxiety. BMC Oral Health.

[25] Zampino C., Ficacci R., Checcacci M., Franciolini F., Catacuzzeno L. (2018). Pain Control by Proprioceptive and Exteroceptive Stimulation at the Trigeminal Level. Front. Physiol..

[26] Malik A. (2015). Technique tips—Distraction anaesthesia: Applying the gate control theory in delivering painless anaesthesia. Dent. Update.

[27] Jain N., Juneja P., Masih U., Bhushan A.K.B., Bhaduaria U.S., Badjatya K. (2021). Efficacy of external cold and a vibrating device in reducing pain and anxiety during local anaesthesia. J. Fam. Med. Prim. Care.

[28] Putrino A., Abed M.R., Marinelli E., Zaami S. (2023). Pain Relief in Dental Local Anaesthesia with Vibrational Devices: Much Ado about Nothing? A Scoping Review. J. Clin. Med..

[29] Singh R., Gupta N., Gambhir N. (2022). Comparative Evaluation of Reduction in Pain Perception Using 5% Topical LA vs. Freezed Cone as a Preparatory Agent for Intraoral Injection in Children and Effect of VRD as Distraction Technique. Int. J. Clin. Pediatr. Dent..

[30] Karuppiah M., Balamurugan S.R., Rajashekaran S., Chowdhary N., Vundala R.R., Shaji N.E. (2024). Evaluation of Effect of Distraction Techniques Using Virtual Reality and Eight-dimension Audio Analgesia Methods on Pain Perception and Anxiety Levels in Children During Restorative Procedures: A Comparative In Vivo Study. Int. J. Clin. Pediatr. Dent..

[31] Bahrololoomi Z., Vaez K., Yekani A.H., Irannezhad M., Parvizi Z. (2025). Comparative efficacy of virtual reality headset and auditory distraction for reducing anxiety and pain during inferior alveolar nerve block in 7-10-year-old children: A clinical trial. BMC Pediatr..

[32] Gurav K.M., Kulkarni N., Shetty V., Vinay V., Borade P., Ghadge S., Bhor K. (2022). Effectiveness of Audio and Audio-Visual Distraction Aids for Management of Pain and Anxiety in Children and Adults Undergoing Dental Treatment—A Systematic Review And Meta-Analysis. J. Clin. Pediatr. Dent..

[33] Juárez-López M.L.A., Marin-Miranda M., Lavalle-Carrasco J., Pierdant A., Sánchez-Pérez L., Molina-Frechero N. (2022). Association of age and temperamental traits with children’s behaviour during dental treatment. Int. J. Environ. Res. Public Health.

[34] Ashley P.F., Chaudhary M., Lourenço-Matharu L. (2018). Sedation of children undergoing dental treatment. Cochrane Database Syst. Rev..

[35] Garrocho-Rangel A., Ibarra-Gutiérrez E., Rosales-Bérber M., Esquivel-Hernández R., Esparza-Villalpando V., Pozos-Guillén A. (2018). A video eyeglasses/earphones system as distracting method during dental treatment in children: A crossover randomised and controlled clinical trial. Eur. J. Paediatr. Dent..

[36] Barreiros D., de Oliveira D.S.B., de Queiroz A.M., da Silva R.A.B., de Paula-Silva F.W.G., Küchler E.C. (2018). Audiovisual distraction methods for anxiety in children during dental treatment: A systematic review and meta-analysis. J. Indian. Soc. Pedod. Prev. Dent..

[37] Liu Y., Gu Z., Wang Y., Wu Q., Chen V., Xu X., Zhou X. (2019). Effect of audiovisual distraction on the management of dental anxiety in children: A systematic review. Int. J. Paediatr. Dent..

[38] Kong X., Song N., Chen L., Li Y. (2024). Non-pharmacological interventions for reducing dental anxiety in pediatric dentistry: A network meta-analysis. BMC Oral Health.

[39] Putrino A., Cassetta M., Raso M., Altieri F., Brilli D., Mezio M., Circosta F., Zaami S., Marinelli E. (2024). Clinical Applications, Legal Considerations and Implementation Challenges of Smartphone-Based Thermography: A Scoping Review. J. Clin. Med..

[40] Gasparro R., Leonetti G., Riccio M., Irace A., Sammartino G., Blasi A., Scandurra C., Maldonato N.M., Sammartino P., Marenzi G. (2021). Thermography as a method to detect dental anxiety in Oral Surgery. Appl. Sci..

